# Atorvastatin inhibits the expression of RAGE induced by advanced glycation end products on aortas in healthy Sprague–Dawley rats

**DOI:** 10.1186/1758-5996-6-102

**Published:** 2014-09-23

**Authors:** Lei Xu, Panpan Zang, Bo Feng, Qiaohui Qian

**Affiliations:** Department of Endocriology and Metabolic Disease, East Hospital, Tongji University School of Medicine, Shanghai, 200120 China; Department of Endocriology, Shanghai Ninth People’s Hospital Affiliated Shanghai Jiaotong University School of Medicine, Shanghai, 200120 China; Department of Endocriology, Shanghai Zhoupu Hospital, Shanghai, 200120 China

**Keywords:** AGEs, RAGE, Atorvastatin, Atherosclerosis

## Abstract

**Background:**

Atorvastatin can downregulate the expression of receptor for advanced glycation end products (RAGE) in the aortas of diabetic rats. However, its effect on healthy rats remains unclear. The aim of this study was to observe the direct impact of atorvastatin on advanced glycation end products- (AGEs) induced RAGE expression in healthy Sprague Dawley (SD) rats.

**Methods:**

SD rats received AGE-BSA (20 mg/kg/day or 40 mg/kg/day), dual treatment (AGE-BSA 40 mg/kg/day and atorvastatin 20 mg/kg/day) or no treatment for 12 and 24 weeks, respectively. The deposition of AGEs and expression of RAGE in the animals’ aortas were assessed by Quantitative RT-PCR, immunohistochemistry, and western-blot tests. Serum levels of AGEs were measured using ELISA.

**Results:**

AGE-BSA upregulated the serum level of AGEs, deposition of AGEs, and expression of RAGE in aortas in a time- and dose-dependent way that can accelerate the development and progression of atherosclerosis. These upregulations could be significantly attenuated by atorvastatin in the absence of its lipid-lowering effects. These data provide further evidence for the novo mechanism of atorvastatin’s pleiotropic effect.

**Conclusion:**

Atorvastatin has a direct inhibitory effect on AGEs-RAGE expression in healthy SD rats. These potential pleiotropic vasculoprotective effects are independent of effects on glucose and lipid control.

## Background

Hyperglycaemia drives non-enzymatic glycation and oxidation of proteins and lipids, which enhances irreversible formation of advanced glycation end products (AGEs) [1]. Accumulation of preformed AGEs in the vessel wall has been shown to promote diabetic vascular diseases [2]. The receptor for AGEs (RAGE) is a multi-ligand receptor that mediates the action of AGEs.

RAGE was initially isolated from the lung, but is also expressed on the surface of vascular endothelial cells, smooth muscle cells, and macrophages [3]. Accumulating evidence suggests that RAGE plays a pivotal role in promoting inflammatory processes and endothelial activation, which accelerates atherosclerosis in patients with diabetes [4]. Binding of AGEs to RAGE activates multiple intracellular signalling pathways, including p21ras, which recruits downstream targets such as mitogen-activated protein kinases (MAPK) and activates nuclear factor kappaB (NF-κB) [5, 6]. The AGEs-RAGE interaction augments inflammatory responses and leads to vascular dysfunction and monocyte activation [7]. Diabetes-associated atherosclerotic lesions display increased accumulation of RAGE ligands and enhanced expression of RAGE [4, 8].

3-Hydroxy-3-methylglutaryl CoA (HMG-CoA) reductase inhibitors, also known as statins, are commonly used for the treatment of dyslipidaemia. A large number of studies have demonstrated that statin therapy is associated with a reduction in cardiovascular events in people both with and without diabetes [9–12]. While a reduction in low-density lipoprotein- cholesterol (LDL-C) was observed in many of these studies, it is now considered that statins also mediate pleiotropic antiatherogenic effects that are partially independent of their cholesterol-lowering effects and may contribute to their efficacy in reducing cardiovascular events. These cholesterol-independent effects include improving endothelial function, attenuating vascular and myocardial remodelling, inhibiting vascular inflammation and oxidation, and stabilizing atherosclerotic plaques [13–15].

In our previous study, we found that atorvastatin can significantly downregulate the expression of RAGE in aorta of diabetic Goto Kakisaki (GK) rats [16]. Those data demonstrated a novel “pleiotropic” activity of atorvastatin in reducing the risk of cardiovascular diseases by targeting RAGE expression. However, no work has been done on the direct effects of atorvastatin on the formation and deposition of AGEs and the expression of RAGE in healthy animal models. In the present study, we provided novel data that suggested atorvastatin could decrease the AGEs-induced increase in serum level of AGEs and suppress the expression of RAGE of aorta in Sprague Dawley (SD) rat models in euglycemic conditions. These results will further confirm the pleiotropic activity of atorvastatin by targeting RAGE.

## Methods

### Preparation of AGEs

Bovine serum albumin (BSA) and D-glucose were dissolved in PBS (pH 7.2–7.4): the final concentrations of BSA and D-glucose were 5 g/L and 50 mmol/L, respectively. EDTA was added to a final concentration of 0.5 mmol/L to reduce oxidation. Penicillin (100 U/L) and streptomycin (100 μg/ml) were added to the reaction mixture to prevent bacterial contamination. The reaction mixture was filtered through 0.22 μm filter and then incubated at 37°C for 12 weeks.

At the end of the incubation period, the reaction mixture was dialyzed against sterilized PBS (pH 7.2–7.4) to remove the unconjugated glucose. The glucose level in the dialyzate was <0.03 mmol/L. The reaction mixture was measured in a fluorospectrophotometer with an excitation wave of 370 nm, and the maximum absorption peak was measured at 440 nm to verify that the mixture was glycated-BSA. Finally, the glycated-BSA was freeze-dried and stored at 4°C.

### Animal experiment and sample collection

Male SD rats (SLRC Laboratory Animal centre, Shanghai, China) weighing 200–250 g (starting age 8 weeks) were divided into five groups. Group A ate standard chow (n = 10). Group B had a high-fat diet containing 69.13% standard chow to which we added 1.37% cholesterol, 0.5% bile salts, 9% sugar, and 20% lard. (n = 10). Group C ate the high-fat diet plus AGEs with glycated-BSA 20 mg/kg body weight per day through intraperitoneal injection (n = 10). Group D ate the high-fat diet plus AGEs with glycated-BSA 40 mg/kg body weight per day through intraperitoneal injection (n = 10). Group E consumed the high-fat diet plus AGEs with glycated-BSA 40 mg/kg body weight per day through intraperitoneal injection and atorvastatin (Lipitor, Pfizer Ireland Pharmaceuticals) 20 mg/kg/day, through intragastric administration (n = 10).

We chose SD rats as the animal models because of their normal glucose levels without obvious insulin resistance compared with the Goto Kakisaki (GK) rats used in our previous study. We chose the dose of atorvastatin mainly on the basis of our previous study [16]. As one of a series of experiments, we chose the same dose of atorvastatin for comparisons. It is higher than the human dose (1.1 mg/kg per day) recommended for treatment of hypercholesterolemia [17], but consistent with the pharmacokinetic data indicating a higher metabolic rate of the drug in rodents [18].

Body weight was measured weekly. At the end of the 12th and the 24th week, five rats per group were anaesthetised by an intraperitoneal injection of pentobarbitone sodium (100 mg/kg body weight) (Euthatal; Sigma-Aldrich, Castle Hill, NSW, Australia). Blood was collected from the left ventricle and centrifuged (6,000 × g), and plasma samples were stored at −20°C for subsequent analysis. Animals were sacrificed and the aortas were rapidly dissected and snap frozen in liquid nitrogen and stored at −80°C or stored in buffered formalin (10%, vol./vol.) for subsequent measurement. All animal experiments were conducted according to the protocol approved by the Animal Committee of the Animal Center of East Hospital, Tongji University.

### Measurement of serum glucose, lipids, and AGEs

Serum glucose levels were measured by the glucose oxidase method (Sigma, MO). Total cholesterol, triglycerides, LDL-C and high-density lipoprotein-cholesterol (HDL-C) levels were measured using a kit from Sigma Diagnostics. The serum levels of AGEs were determined using commercially available enzyme linked immunosorbent assay (ELISA) kits (Xitang Bio Technology Co, Shanghai, China) according to the manufacturer’s instructions.

### Quantitative real-time PCR analysis

Total RNA was isolated with the trizol method and depurated with an RNAeasy kit (Invitrogen, CA). RNA was stored at −80°C until reverse transcription was performed. An aliquot (1 μg) of extracted RNA was reverse-transcribed into the first strand of complementary DNA (cDNA) at 42°C for 40 min, using 100 U/ml reverse -transcriptase (Takara Biochemicals, Shiga, Japan) and 0.1 μM of oligo (dt)- adapter primer (Takara) in a 50 ul reaction mixture.

Real-time polymerase chain reaction (PCR) was carried out with an ABI Prism 7000 Real-Time PCR system, using the DNA-binding dye SYBER Green I for the detection of PCR products. The reaction mixture (RT-PCR kit, Takara) contained 12.5 μl Premix Ex Tag, 2.5 μl SYBER Green I, custom-synthesized primers, ROX reference dye, cDNA (equivalent to 20 ng total RNA) to give a final reaction volume of 25 μl. Primers were as follows:

GAPDH: sense 5’AGAGGAGAGGAAGGCCCCAGA 3’, antisense 5’GGCAAGGTGGGGTTATACAGG 3’;

RAGE: sense 5’GACAACTTTGGCATCGTGGA 3’, antisense 5’ATGCAGGGATGATGTTCTGG 3’.

The PCR settings were as follows: initial denaturation of 30 s at 95°C, followed by 40 cycles of amplification for 5 s at 95°C and 34 s at 60°C, with subsequent melting curve analysis increasing the temperature from 60°C to 95°C. To quantify RAGE gene expression, the RAGE mRNA level was normalized by internal GADPH mRNA.

### Hematoxylin and eosin (HE) staining and immunohistochemistry

Thoracic aorta sections were rehydrated and placed in Gill’s c2 hematoxylin (Medical reagent company, Shanghai, China) and then transferred to an eosin Y solution (Medical reagent company, Shanghai, China). After staining, the sections were dehydrated through the alcohol series back to xylene and a coverslip was mounted to each slide with Permount.

Immunostaining of AGEs and RAGE was performed using the Vectastain Elite ABC Mouse/Rabbit IgG kit based on an avidin/biotin/peroxidase system (Boster Biotechnology Co. Ltd, Wuhan, China) according to the manufacturer’s instructions. Briefly, the sections were incubated with 5% bovine serum albumin in PBS at room temperature for 30 min to block nonspecific binding of the second antibody and then reacted with the primary antibodies against AGEs (1:50, Santa Cruz Biotechnology) or RAGE (1:50, Santa Cruz Biotechnology) at 4°C overnight. Then they were exposed to the biotinylated secondary antibody at room temperature for 30 min. After washing in PBS, the specimens were incubated with an avidin-biotin-peroxidase complex at room temperature for 30 min. Deposition was visualized by treating the sections with DAB. The sections were viewed and photographed with an Olympus XI 70 microscope and digital imaging system (Olympus Optical, Tokyo, Japan). Image Pro Plus 6.0 was used to analyse the average of optical density (AOD) in the positive staining area.

### Western blot analysis

Aortas were dissected and used to analyse protein levels by western blot. The lysates (30 μg protein) were separated by 10% SDS-PAGE, transferred to a PVDF membrane (Millipore), blocked with 5% non-fat dry milk for 60 min, and probed overnight with antibodies at 4°C. The blots were incubated with HRP-conjugated anti-IgG for 1 h at 37°C. We detected protein bands using chemiluminescence reagent Amersham ECL plus (GE Healthcare, Buckinghamshire, UK). Relative intensities of protein bands were analysed by Gel-Pro Analyzer software. Antibodies against RAGE (1:1000 dilution) and β-actin (1:1000 dilution) were purchased from Cell Signaling, USA.

### Statistical analysis

All data were expressed as mean ± SD. Kruskal-Wallis one-way analysis of variance was used to assess the differences between groups using SPSS 13.0 software. We considered values of P < 0.05 statistically significant.

## Results

### Metabolic parameters

At the end of the 12th week, the body weights of rats in Groups C and D were higher as compared to other groups, but that changes were statistically insignificant. But at the end of the 24th week, a significant increase in body weight in Group D was observed (*P* < 0.05). Serum glucose levels and LDL-C levels were not different among all five groups at both the 12th week and the 24th week (Table 1).

**Table 1:** **Metabolic parameters of SD rats in different groups**

	Body weight		Serum glucose		LDL-c	
	12th week	24th week	12th week	24th week	12th week	24th week
Group A	603.40 ± 37.72	750.20 ± 40.24	5.62 ± 0.28	6.29 ± 0.24	2.78 ± 0.62	1.81 ± 0.38
Group B	621.25 ± 46.82	720.36 ± 33.69	6.18 ± 0.67	5.79 ± 0.66	1.74 ± 0.58	1.79 ± 0.52
Group C	630.13 ± 28.41	784.35 ± 38.53	5.98 ± 1.16	5.72 ± 0.69	2.08 ± 0.37	2.24 ± 0.42
Group D	626.38 ± 32.32	806.61 ± 48.25*	5.95 ± 0.92	6.24 ± 0.75	2.04 ± 1.01	2.08 ± 0.85
Group E	616.13 ± 42.38	771.5 ± 40.08	5.62 ± 0.90	6.20 ± 0.28	2.74 ± 0.29	2.72 ± 0.36

Group A: absolute controls, (n = 10); Group B: high fat diet (n = 10); Group C: high fat diet plus AGEs with glycated-BSA 20 mg/kg body weight (n = 10); Group D: high fat diet plus AGEs with glycated-BSA 40 mg/kg (n = 10)#Group E: high fat diet plus AGEs with glycated-BSA 40 mg/kg and atorvastatin. Each value represents the mean ± S.D. * = p < 0.05 vs. other groups at the end of 24th week.

### The change of serum AGEs levels

**Figure 1:**
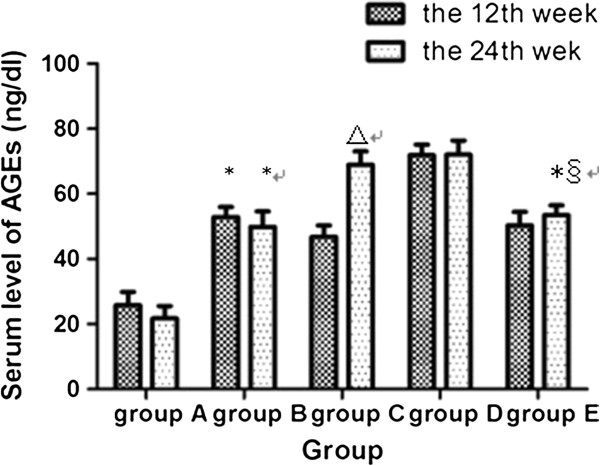
**Serum AGEs levels in different groups at the end of the 12th week and the 24th week respectively.** *compared with group A, P < 0.05; △compared with group A and group B, P < 0.05; §compared with group D, P < 0.05. Group A: absolute controls, (n = 10); Group B: high fat diet (n = 10); Group C: high fat diet plus AGEs with glycated-BSA 20 mg/kg body weight (n = 10); Group D: high fat diet plus AGEs with glycated-BSA 40 mg/kg (n = 10)#Group E: high fat diet plus AGEs with glycated-BSA 40 mg/kg and atorvastatin.

Compared with Group A, there was significant increase (about double) in the serum AGEs levels in Group B at the 12th and the 24th weeks (52.85 ± 3.08 vs 25.78 ± 4.09 at the 12th week,49.91 ± 4.70 vs 21.69 ± 3.88 at the 24th week,*P* < 0.05). AGEs-BSA treatment induced a further increase compared with Group B, and at the 24th week, the high-dose AGEs-BSA treated group (Group D) had the highest level of all groups with statistical significance, compared to Group A (about 3.3 times as much) (72.02 ± 4.37 vs 21.69 ± 3.88,*P =* 0.002) and Group B (about 1.4 times) (72.02 ± 4.37 vs 49.91 ± 4.70,*P =* 0.009). Atorvastatin treatment reduced the serum AGEs level (Group E) compared with group D by a 26% change with statistical significance at the 24th week (53.43 ± 3.05 vs 72.02 ± 4.37,*P* = 0.013). AGEs levels in Group E were not significantly different compared with Group B, but still higher than Group A about double (53.43 ± 3.05 vs 21.69 ± 3.88, *P* = 0.006) (Figure 1).

### Accumulation of AGEs in aortic wall

**Figure 2:**
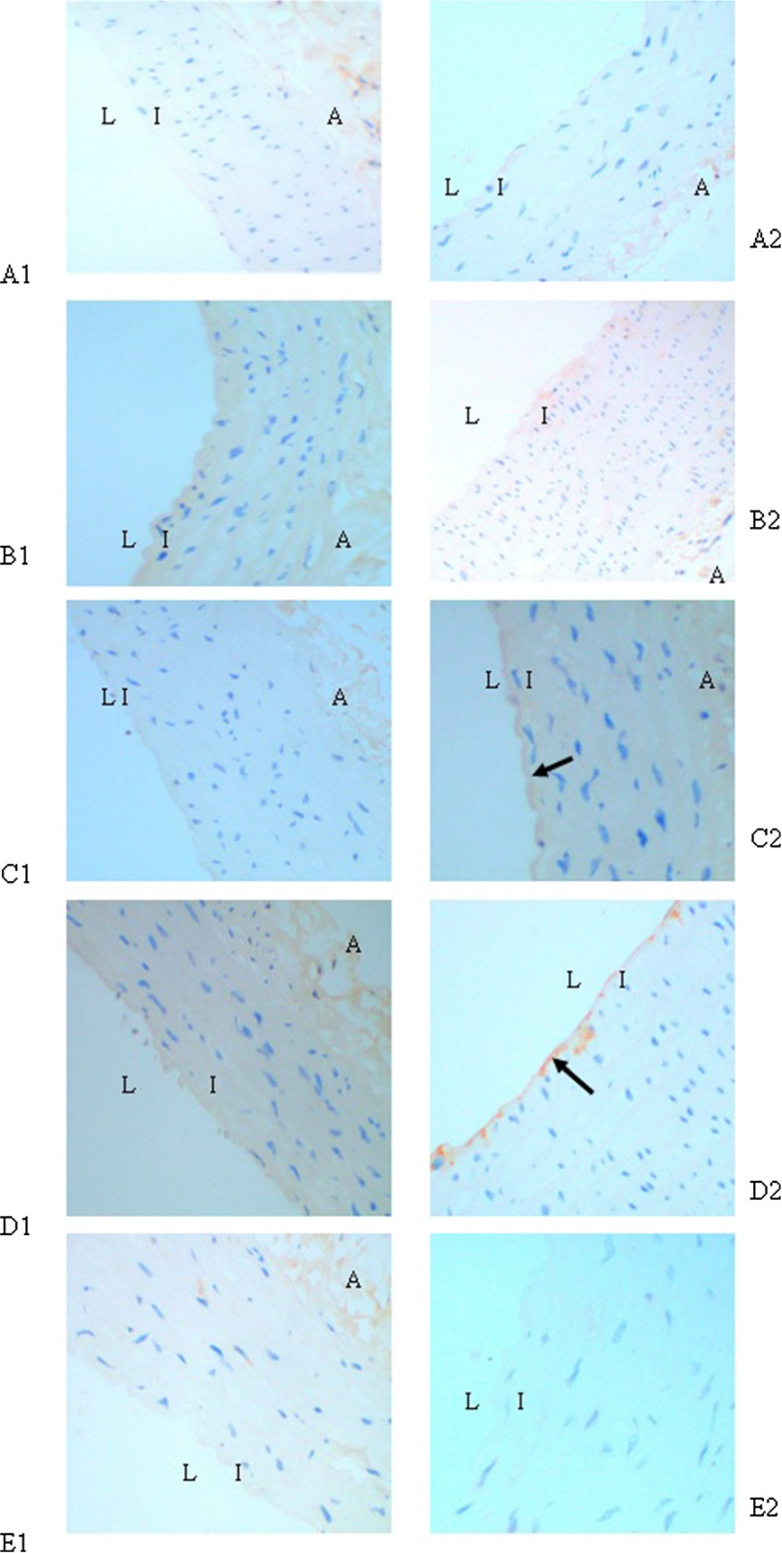
**Immunohistochemical staining of AGEs in aortic wall. A1-E1**, Group A-Group E at the end of the 12th week; **A2-E2**, Group A-Group E at the end of the 24th week. The presence of antigen is indicated by a red-brownish color which was labeled by arrows. Immunoreactivity was positive in the intima. Original magnification is × 200 for all photographs. L: lumen; I: intima; A: adventitia.

The presence of AGEs in aortic walls was immunohistochemically examined in the tissue specimens of the aortas from the five experimental groups. There was no obvious positive immunostaining of AGEs in each group at the 12th week (Figure 2 A1-E1). At the 24th week, immunostaining of AGEs was obvious in Groups C and D (Figure 2 C2-D2), especially in Group D (Figure 2 D2). Atorvastatin attenuated AGEs accumulation significantly compared with Group D (Figure 2 E2).

**Figure 3:**
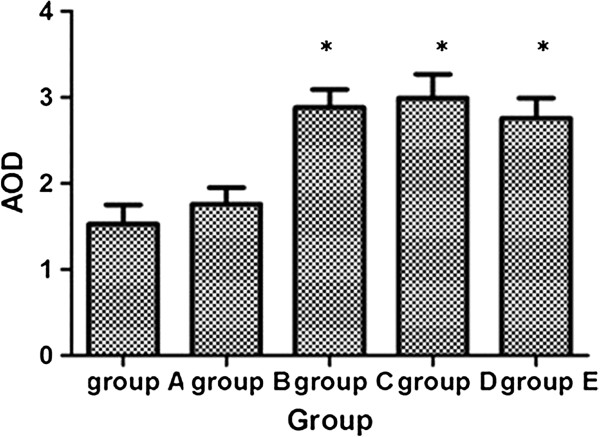
**AOD of the positive staining area of AGEs in different groups at the end of the 24th week.** *compared with group A and group B, P < 0.05.

Then the AOD in the positive staining area was analysed by software. At the 24th week, the accumulation of AGEs had a further increase in Group C and Group D (about double) with statistical significance, compared with Group A (2.88 ± 0.21 vs 1.53 ± 0.22,2.99 ± 0.28 vs 1.53 ± 0.22,respectively; *P* < 0.001) and about 1.6 times that of Group B (2.88 ± 0.21 vs 1.76 ± 0.19; 2.99 ± 0.28 vs 1.76 ± 0.19,respectively; *P* = 0.001). Atorvastatin reduced the presence of AGEs without statistical significance compared with Group D at the 24th week (Figure 3).

### Expression of RAGE in mRNA levels in the aortic wall

**Figure 4:**
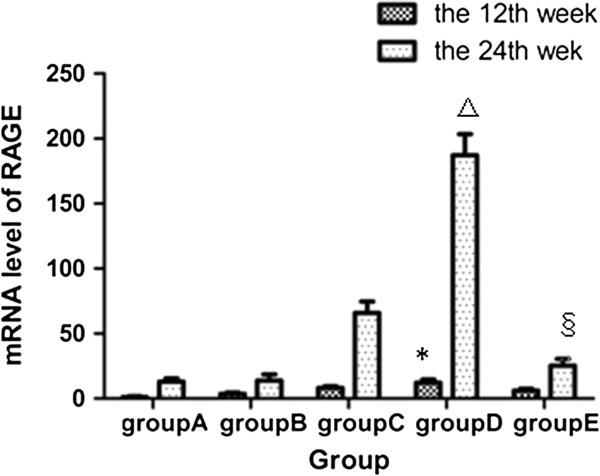
**Expression of RAGE in mRNA level of different groups at the end of the 12th week and the 24th week respectively.** *compared with group B, P < 0.05; △compared with other four groups, P < 0.05; §compared with group C and group D, P < 0.05.

Compared with Group A, the expression of RAGE mRNA was significantly increased in Groups B, C, and D at the 12th week (*P* < 0.01). At the 24th week, RAGE mRNA level in Group D increased around 14 times compared with that in Group A and B (187.16 ± 16.33 vs 13.08 ± 2.34; 187.16 ± 16.33vs 13.87 ± 4.68,respectively; *P* < 0.01) and about 3 times with that in Group C (187.16 ± 16.33 vs 65.90 ± 8.87; *P* = 0.002). In the atorvastatin-treated group, however, RAGE mRNA levels were significantly downregulated to a level comparable to those observed in the normal Group A and high-fat diet Group B at the 24th week (Figure 4).

### Expression of RAGE in protein levels in the aortic wall

**Figure 5:**
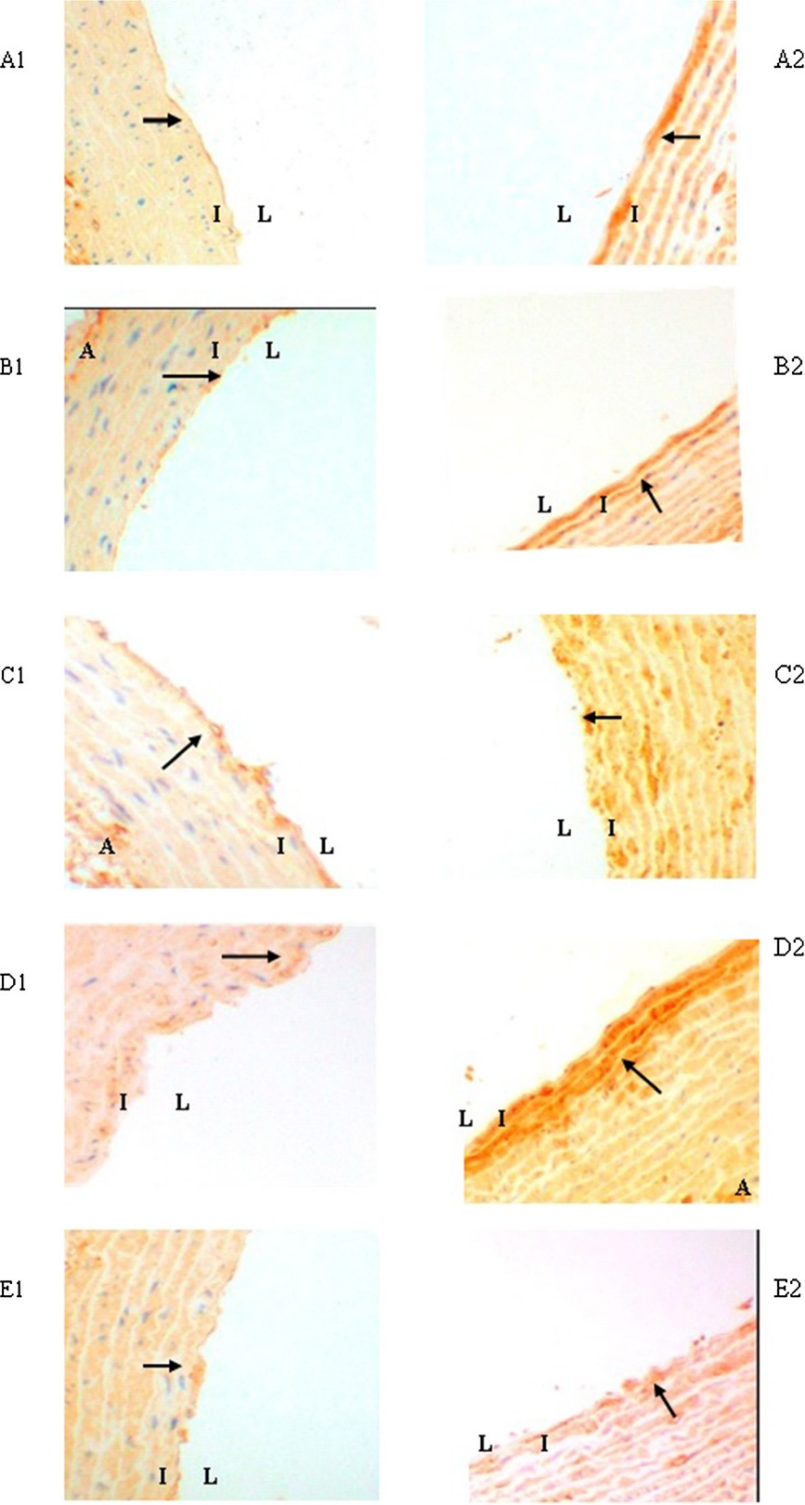
**Expression of RAGE in aortic wall of different groups at the end of the 12** ^**th**^ **week and the 24** ^**th**^ **week by immunohistochemical staining. A1-E1**, Group A-Group E at the end of the 12th week; **A2-E2**, Group A-Group E at the end of the 24th week. The presence of antigen is indicated by a red-brownish color which was labeled by arrows. Original magnification is × 200 for all photographs. L: lumen; I: intima; A: adventitia.

Immunohistochemistry was used to observe expression of RAGE in aortas. Both at the 12th week and the 24th week, the expression of RAGE was higher in Group B than Group A. The AGEs-BSA treated groups (C and D) had higher expressions of RAGE than Group B. At the 24th week, deeper staining and more enlarged positive areas were observed in these five groups than at the 12th week. Atorvastatin reduced the expression of RAGE, compared with Group D (Figure 5).

**Figure 6:**
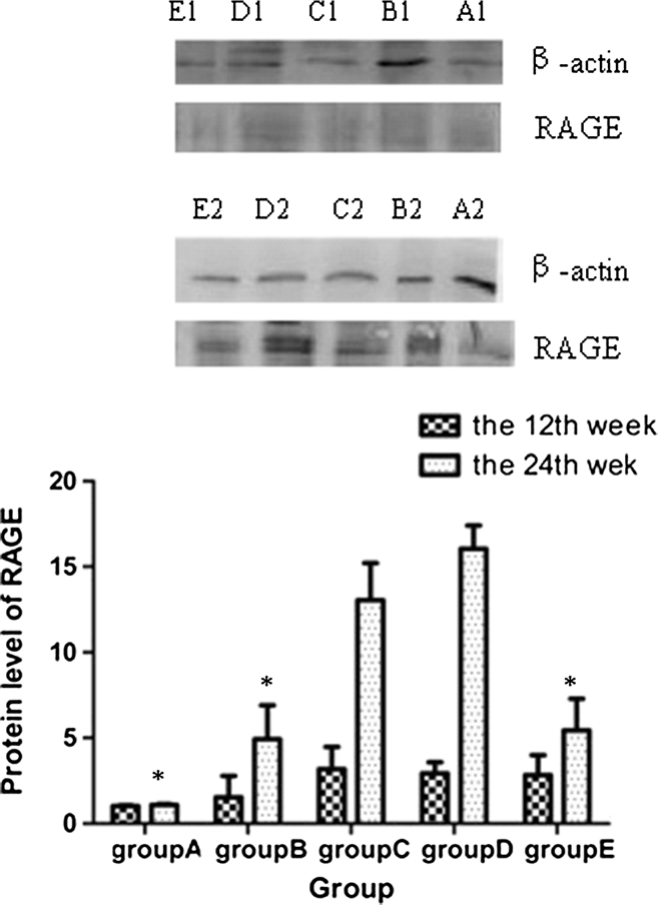
**Expression of RAGE in protein level of different groups at the end of the 12th week and the 24th week respectively.** *compared with group C and group D, P < 0.05.

The quantitative determination of RAGE in aorta was detected by western blot. There were no significant differences among five groups at the 12th week. At the 24th week, the expression of RAGE was significantly higher in Groups C and D than Group A (13.04 ± 2.17 vs 1.07 ± 0.02; *P* = 0.023 and 16.04 ± 1.36 vs 1.07 ± 0.02; *P* = 0.003, respectively) and Group B (13.04 ± 2.17 vs 4.92 ± 1.98; *P* = 0.032 and 16.04 ± 1.36 vs 4.92 ± 1.98; *P* = 0.008, respectively). Atorvastatin significantly suppressed the expression of RAGE at the 24th week with about 66% decrease compared with Group D (5.45 ± 1.84 vs 16.04 ± 1.36,*P* = 0.008) (Figure 6).

### Morphological examination of aortas

The general morphology of aortas in all 5 groups at the 12th week and the 24th week remained relatively constant throughout the study.

## Discussion

Statins may be able to reduce cardiovascular events by mechanisms other than their ability to lower the level of serum lipids, i.e. the “pleiotropic” actions [19, 20]. Furthermore, there is a growing body of evidence that statins can decrease the serum AGEs levels and expression of RAGE in diabetic aortas with atherosclerotic plaque [21–23]. This effect is supposed to be a novel molecular mechanism of statin’s pleiotropic actions.

Previously we have demonstrated, using the diabetic GK rats, that the RAGE expression in diabetic rats was significantly reduced by treatment with atorvastatin independent of glycaemic and lipid control in the very early stage of atherosclerosis, possibly through an epigenetic mechanism [16]. However, nearly all prior studies have been done in diabetic animal models and cannot exclude the effects of interference factors such as the level of blood glucose on the results. Therefore these studies cannot provide exact evidence whether statins can inhibit the expression of RAGE directly in tissues of arteries. Therefore, in present study, we chose healthy SD rats treated with AGEs to simulate the diabetic pathological changes in arteries induced by AGEs to exclude the interference factors and to determine the direct effects of atorvastatin on regulation of AGEs and RAGE.

Although we divided the SD rats into a standard chow group and high fat diet groups and treated some of them by atorvastatin or AGEs, unexpectedly there were still no significant differences in glucose levels and lipid profile levels among these five groups. All the changes in this study were independent from glycaemic and lipid control.

In the present study, serum AGEs levels and the accumulation of AGEs in aortas were significantly increased by AGEs induction in a dose- and time-dependent manner. Concentrations of circulating AGEs correlate to the severity of coronary artery disease and adverse clinical outcomes [24]. Atorvastatin can reduce the serum level of AGEs in a time-dependent and a cholesterol-lowering independent manner; the exact mechanisms are unclear. Oxidative stress participates in the formation of AGEs, which are by themselves a source of free-radical superoxide generation as well [25, 26]. Furthermore, hydroxyl metabolites of atorvastatin have been shown to have anti-oxidative properties [27].

Our previous study suggests that atorvastatin can improve the oxidative stress induced by AGEs. The present findings suggest that atorvastatin may decrease serum AGEs levels via its anti-oxidative property. On the other hand, atorvastatin may reduce the absorption of exogenous AGEs through peritoneal capillary networks and the intestinal canal through its anti-inflammatory and anti-oxidative properties, which can reduce the inflammatory action and oxidative stress of the intestinal wall [28, 29]. This may be another possible mechanism to explain the effect of atorvastatin on decreasing serum AGEs levels.

In spite of the obvious reduction of serum AGEs level, we did not find a significant reduction of accumulation of AGEs in aorta in Group E, treated with atorvastatin, compared with Group D. AGEs slowly degrade and remain for a long time in diabetic vessels even after glycaemic control and oxidative stress conditions have improved [30]. Therefore, the phenomenon of so-called metabolic memory could be explained, in part, by AGEs [31]. In other words, the accumulation of AGEs on aortas may not be easily reversed by short-term statin therapy.

There is a growing body of evidence that RAGE may play a pro-atherogenic role in diabetic arteries [32, 33]. Our data showed that the expression of RAGE on aortas significantly increased in AGEs-treated groups, and this increase correlated with time, course, and concentration of serum AGEs. Atorvastatin could reduce the expression of RAGE on aortas both in the mRNA and protein levels, which is in line with our previous observations [16]. This effect of atorvastatin was partly independent of the reduction of AGEs accumulation on aortas. Excluding other interference factors of hyperglycaemia in our present study, we can speculate that atorvastatin downregulated the expression of RAGE partly by reducing the serum AGEs levels and blocking the AGEs-RAGE positive feedback loop.

The other possible mechanism is that atorvastatin can inhibit gene expression of RAGE directly. There is a growing body of evidence that statins could inhibit plaque RAGE expression in type 2 diabetes [23, 34, 35]. Furthermore, our previous study demonstrated that atorvastatin suppressed the AGE/RAGE pathway by a gene targeting mechanism in diabetic atherosclerosis.

In the present study, we demonstrated that atorvastatin can downregulate RAGE expression in normal conditions without type 2 diabetes or atherosclerosis. These results are not only in accordance with our previous study, but also complement previous results. These data can provide further evidence for the novo mechanism of atorvastatin’s pleiotropic effect.

Notably, the high-fat diet raised the serum level of AGEs and expression of RAGE in spite of a lack of change in the serum level of cholesterol. In our previous study, we found that a high-fat diet could promote the oxidative stress that may increase the formation of AGEs.

In addition, treatment of AGEs can increase the body weight of SD rats, although this has rarely been reported in previous studies, and the specific mechanism was unclear. One study found that weight increases were associated with skeletal muscle immunostaining for AGEs, RAGE, and oxidation injury [36]. The AGEs-RAGE axis, oxidative stress, weight gain, and insulin resistance may correlate with each other, and further study should explore this area.

There was no change in the morphology of the aortas in the five groups, which was in accordance with our previous study [16]. This shows that atorvastatin can downregulate the AGEs-RAGE axis in the very early stages of atherosclerosis, before its therapeutic improvement of the atherosclerotic lesions can be detected histologically. Thus, our data demonstrate there would be more benefits for diabetic patients from using atorvastatin earlier at a full, effective dose.

## Conclusions

The present results demonstrate the direct inhibitory effects of atorvastatin on AGEs-RAGE expression in healthy SD rats. These potential pleiotropic vasculoprotective effects are independent of effects on glucose and lipid control. This study provides strong evidence for the early use of atorvastatin in diabetes-associated atherosclerosis; this agent has the potential to exert superior long-term vasculoprotection. Future studies should be done to determine the exact molecular mechanisms and signalling pathways of the effects of atorvastatin on AGEs-RAGE expression.

## Abbreviations

AGEs: Advanced glycation end products
RAGE: Receptor for advanced glycation end products
MAPK: Mitogen-activated protein kinases
NF-κB: Nuclear factor kappaB
HMG-CoA: 3-Hydroxy-3-methylglutaryl CoA
LDL-C: Low-density lipoprotein- cholesterol
GK: Goto Kakisaki
SD: Sprague Dawley
BSA: Bovin serum albumin
HDL-C: High density lipoprotein-cholesterol
ELISA: Enzyme linked immunosorbent assay
HE: Hematoxylin and eosin
AOD: Average of optical density.
